# Survival Rates of Human Immunodeficiency Virus and *Tuberculosis* Co-Infected Patients

**DOI:** 10.5812/jjm.10565

**Published:** 2014-06-01

**Authors:** Ghodratollah Roshanaei, Masoud Sabouri Ghannad, Mohammad Saatchi, Salman Khazaei, Mohammad Mirzaei

**Affiliations:** 1 Modeling of Noncommunicable Diseases Research Center, Department of Biostatistics and Epidemiology, School of Public Health, Hamadan University of Medical Sciences, Hamadan, IR Iran; 2 Department of Microbiology, Research Center for Molecular Medicine, Faculty of Medicine, Hamadan University of Medical Sciences, Hamadan, IR Iran; 3 Department of Biostatistics and Epidemiology, School of Public Health, Hamadan University of Medical Sciences, Hamadan, IR Iran; 4 Department of Health Services, Hamadan University of Medical Sciences, Hamedan, IR Iran

**Keywords:** Tuberculosis, Complications, Survival Rate

## Abstract

**Background:**

At present, limited clinical data is available regarding survival rates of patients co-infected with human immunodeficiency virus (HIV)/*tuberculosis* (TB) in developing countries.

**Objectives:**

The present study aimed to evaluate the effect of HIV infection on the survival chances of active TB adults who disclosed their symptoms of TB in this part of Iran.

**Patients and Methods:**

The records and data of 807 patients only infected with TB and 21 co-infected patients with HIV/TB, who were admitted to primary health care units in Iran, were evaluated. Their survival time was analyzed using the Kaplan-Meier Estimator, Log-rank test and SPSS version 16.

**Results:**

Cox regression analysis showed that co-infection with HIV significantly affects the survival rate of TB patients so that the rate of death was 20.7 (8.1-53) times more than TB infected patients alone. Also, married patients with tuberculosis were 2.7 times more at risk of death than single subjects. We also confirmed that in HIV/TB positive patients, married individuals were more prone to death than single subjects (P value < 0.001).

**Conclusions:**

Our results denote the need to progress diagnostic and preventive measures in this part of Iran.

## 1. Background

Human Immunodeficiency Virus (HIV), which can lead to acquired immunodeficiency syndrome (AIDS)-related diseases, has been reported as the cause of death in almost 30 million people (1). Further work has also revealed that *Mycobacterium tuberculosis* (MTB) is influencial in public health and is responsible for the most common life-threatening opportunistic infection in patients (2) with HIV worldwide (3). Globally, tuberculosis kills one-third of patients co-infected with AIDS (4). It has been estimated that 14 million people are dually infected worldwide. Tuberculosis is responsible for about 26% of AIDS-related deaths, with 99% occurring in developing countries (5). Thus far, regarding survival rates, limited clinical data is available for HIV+TB+ patients in developing countries (6). 

Increased spread of TB is still the subject of considerable debate and represents a high priority goal in order to attain better insight into modeling of co-infection of HIV+TB+ and the multiple factors driving the survival time of this group of patients in other parts of Iran and also in regional countries. Based on a report from Iran between 1992 and 1999, the rate of TB has reduced nationwide which may be indicative of successful development of public health policies (7). Official figures from the Ministry of Health and Medical Education in Iran indicated that in 2010, the estimated number of people living with old and new cases of TB was 10485 in Iran which included 326 (approximately 2.2%) HIV infected patients (8). Based on an official report from Iran, the country has registered 24290 HIV-positive citizens until April 2012 (9).

## 2. Objectives

The present study aimed to evaluate the possible variables that may influence the survival time of HIV+TB+ patients. The results will show the importance of treatment of higher priority patients.

## 3. Patients and Methods

This was a retrospective descriptive study. Records and data were collected from 807 patients with only TB and 21 co-infected patients with HIV+TB+ who were admitted to primary health care units between 2005-2010 in Hamadan, a western province in Iran. The demographic data included sex, residential area, age, marital status, job and the clinical features included site of TB, AIDS stage based on that proposed by the world health organization (WHO) staging system from stage 1 (stages 1 to 3 are included as non AIDS stages) to stage 4 (AIDS) (10). The usage of multiple drugs that act on different viral targets, known as Highly Active Antiretroviral Therapy (HAART) and the results of sputum smear of 827 patients who had been referred to primary health care units and were co-infected with TB/HIV, were recorded in the questionnaires. The diagnostic criteria for TB included cough, fever, hemoptysis, weight loss, loss of appetite and a positive Mantoux tuberculin skin test (TST). Our study was based on national protocols for the diagnosis of tuberculosis, which included the presence of one positive acid-fast-bacilli (AFB) sputum smear plus radiological evidence, which may confirm the possibility of pulmonary TB or two positive sputum smear tests, which often indicate TB disease.

In primary health care units, HIV diagnosis was firstly based on two positive ELISA tests and then a confirmatory test using the western blotting method for the positive ELISA cases. Some of the design for this study was partially based on clinical history and additional exams including HIV-ELISA, hemoglobin, hematocrit and also the Directly Observed Treatment Short-course (DOTS) strategy recommended by WHO (11). Cases with extrapulmonary TB were diagnosed based on amplification of mycobacterial nucleic acid using Polymerase Chain Reaction (PCR) with higher sensitivity and specificity than acid-fast bacilli (AFB) smear of biological specimens. Co-infection of HIV was detected in twenty-one patients. 

We analyzed and described the demographic data, clinical characteristics and treatment and survival time of HIV patients co-infected with TB. In the current research, survival time analysis was performed via modeling of data, which included the period from the time of initiation of an event until death. The phrase “after initiation of symptoms” refers to departure from standard function that is felt by the patient but cannot be measured. The phrase "after diagnosis of infection" refers to diagnostic laboratory infections, which are confirmed by several methods. Furthermore, the phrase "after initiation of treatment" refers to beginning of something considered as a choice for treatment. 

The survival time was analyzed with the Kaplan-Meier Estimator and Log-rank test to verify statistical significance. To assess the factors that effect survival rate, the logistic regression method was used and also chi-square test was performed to evaluate the relationship between variables. A P value < 0.05 was considered significant. The data were recorded using the TB Register software and the entire data was analyzed by the Social Sciences Statistical Software (SPSS) version 16.

## 4. Results

Amongst the patients infected with TB, 394 (48.8%) were male and 413 (51.2%) were female. Regarding the age of the patients, 457 of the patients were over 50 years old (57%) and 350 (3%) were under 50 years old. Regarding residency status, 495 (61.3%) patients lived in urban regions and 312 (38.7%) lived in rural areas. Considering all the HIV-TB+ patients, 736 (91.1) cases were treated, while 71 (8.9%) patients died. In contrast, from the HIV+TB+ patients 7 (33.3) cases survived, while 14 (66.7) cases died. In this study, out of 21 patients co-infected with HIV+TB+, 20 (95.2%) were male and one (4.8%) was female. Amongst the HIV+TB+ patients, 18 (85.7%) were urban residents and three (14.3) were rural inhabitants. 

With regards to patients co-infected with HIV+TB+, there was no patient more than 50 years old and most cases (71.4%) belonged to the 31-40 years old group. Considering the stage of disease, 16 patients (76.2%) were at the AIDS stage and five patients (23.8%) were at the non-AIDS stage. Out of the 21 patients, 12 (57.1%) patients started highly active antiretroviral therapy (HAART) while 9 (42.9%) patients did not use medication for the treatment of AIDS. There was a significant difference in the survival rate of HIV+TB+ co-infected patients and patients infected with TB alone (P value < 0.001). Moreover, there was a significant difference in the survival rate of various categories of (after initiation of symptoms, after diagnosis of infection and after initiation of treatment) HIV+TB+ co-infected patients compared to TB infected patients (P value < 0.001), so that the survival rate of HIV+TB+ co-infected patients was less than TB infected patients. 

Log-rank analysis of survival showed no significant difference in survival rates of rural and urban residents co-infected with HIV+TB+ (P value < 0.001) in comparison to patients infected with TB alone (P value = 0.38). Based on the results, sex, residential area, HIV affliction and age were important parameters of HIV+TB+ patients so that male gender (P value < 0.001), residents of urban areas (P value = 0.02) and 31-50 years old age group (P value = 0.001) were more prone to risk of HIV infection (P value < 0.001). Also, HIV+TB+ patients were more likely to die than TB+ HIV- patients (P value < 0.001) (not shown).

The current results indicated that in HIV-TB+ patients, age, sex and site of TB were significant factors in mortality of patients, confirming that there is a greater death rate in young patients compared to older individuals (P value < 0.001). Moreover, the death rate of men was about two times greater than that of women. Also, the risk of death in patients with pulmonary TB was two times more than extra pulmonary cases (P value < 0.03) (Table 1). From Table 2, it can be concluded that married patients were more susceptible to death than to singles (P value < 0.001). Other factors including sex, residential area, age, job, receiving or not receiving HAART, AIDS stage and sputum smear test did not have any significant effect on the patients' survival (Table 2).

**Table 1.** The Relationship of Various Factors With Mortality in HIV-TB+ Patients

	Vital Status			P Value
	Survived	Died	Total	
**Sex**				0.02 ^a^
Male	350 (88.8)	44 (11.2)	394 (100.0)	
Female	386 (93.5)	27 (6.5)	413 (100.0)	
**Residential area**				0.52
Urban	454 (91.7)	41 (8.3)	495 (100.0)	
Rural	282 (90.4)	30 (9.6)	312 (100.0)	
**Age**				< 0.001 ^a^
≤ 10	8 (80.0)	2 (20.0)	10 (100.0)	
20-11	48 (96.0)	2 (4.0)	50 (100.0)	
21-30	88 (97.8)	2 (2.2)	90 (100.0)	
31-40	107 (93.0)	8 (7.0)	115 (100.0)	
41-50	81 (95.3)	4 (4.7)	85 (100.0)	
60-51	89 (95.7)	4 (4.3)	93 (100.0)	
70-61	97 (89.8)	11 (10.2)	108 (100.0)	
80-71	159 (89.8)	18 (10.2)	177 (100.0)	
> 81	59 (74.7)	20 (25.3)	79 (100.0)	
**Site of TB**				0.03 ^a^
Pulmonary	500 (89.8)	57 (10.2)	557 (100.0)	
Extra pulmonary	236 (94.4)	14 (5.6)	250 (100.0)	
**Prison**				0.1
Negative	723 (91.1)	68 (8.9)	794 (100.0)	
Positive	10 (100.0)	3 (0.0)	13 (100.0)	

^a^ P value < 0.05 considered as significant.

**Table 2.** The Relationship of Various Factors With Mortality in HIV+TB+ Patients ^a^

	HIV+TB+			P Value
	Survived	Died	Total	
**Sex**				0.15
Male	6 (30)	14 (70)	20 (100)	
Female	1 (100)	0	1 (100)	
**Residential area**				1
Urban	6 (33.3)	12 (66.7)	20 (100)	
Rural	1 (33.3)	2 (66.7)	1 (100)	
**Age**				0.12
21-30	0	1 (100)	1 (100)	
31-40	7 (46.7)	8 (53.3)	15 (100)	
41-50	0	5 (100)	5 (100)	
**Marital status**				0.01 ^b^
Single	5 (71.4)	2 (28.6)	7 (100)	
Married	2 (14.3)	12 (85.7)	14 (100)	
**Job**				0.53
Nongovernmental organizations	2 (28.6)	5 (71.4)	7 (100)	
Non employees	3 (33.3)	6 (66. 7)	9 (100)	
Labors	1 (25.0)	3 (75.0)	4 (100)	
Prisoners	1 (100)	0	1 (100)	
**HAART**				1
Negative	3 (33.3)	6 (66.7)	9 (100)	
Positive	4 (33.3)	8 (66.7)	12 (100)	
**AIDS stage**				0.15
Non-AIDS ^c^	3 (60)	2 (40)	5 (100)	
AIDS	4 (25)	12 (75)	16 (100)	
**Sputum smear**				0.35
Negative	3 (25)	9 (75)	12 (100)	
Positive	4 (55.6)	5 (54.4)	9 (100)	

^a^ Abbreviations: HAART, highly active antiretroviral therapy; HIV, human immunodeficiency virus; TB, tuberculosis.

^b^ P value < 0.05 considered as significant.

^c^ From stage one to stage three.

The odds ratio of mortality in married patients with TB was 2.7 (1.7-9.4) with a 95% confidence interval (P value < 0.02) (not shown). This means that married patients with TB were at a 2.7 times greater risk of death compared to single individuals.

## 5. Discussion

In the current study, 2.5% of cases were co-infected with HIV+TB+. This is similar to a report from Georgia with 1.7 to 2.2% co-infection rate (12) and another study from Saudi Arabia indicating that 1.35% of individuals become co-infected per year (13). In contrast, a research from Iran, reported that nearly 24% of Iranian patients with HIV/AIDS were infected with MTB (14). Another study from Nigeria showed that 28.2% of TB patients under study were co-infected with HIV (15). A report performed on different patient groups in St. Petersburg, Russia, confirmed the presence of TB in 77% of HIV-infected cases, in all groups belonging to the Beijing family (16). The discrepancy of our results with some of the previous literature may be because a portion of HIV infected cases, due to cultural and social beliefs may not have referred to related medical centers for treatment (17).

The results of the current study established the significant difference of survival rate between HIV+TB+ co-infected patients and TB infected patients (P value < 0.001). The assessment of risk factors by Cox regression analysis on 828 patients showed that co-infection with HIV significantly affects the survival rate of TB patients so that the rate of death was 20.7 (8.1-53) times more than patients infected with TB alone.

This finding was as expected. In HIV+TB+ patients, married cases were more prone to death than single individuals (P value < 0.01) (Table 2). The latter results provide some interesting insight into the subject of the current research. The reason may due to greater sexual activity of married patients compared to singles, which increases the likelihood of sexually transmitted diseases (STDs). 

Moreover, progress of TB in HIV patients is influenced by both genetic and environmental components that could be an additional explanation for the obtained results. However, the suggested reasons need further exploration in future studies. Also, multivariate analysis confirmed that the married patients with tuberculosis were 2.7 times more at risk for TB death than single cases (P value < 0.02) (not shown). Nevertheless, a research in Thailand showed that married patients were at lower risk of TB death (18). The discrepancy between our findings and the literature remains to be fully understood. Other factors such as sex, residential area, age, job, receiving or lack of HAART, AIDS stage and sputum smear test did not have any significant effect on the survival of HIV+TB+ patients (Table 2). In contrast, a research from Iran showed that sex and marital status were significant factors as well (14).

Furthermore, the current study found significant differences in the survival rate of different categories of (after initiation of symptoms, diagnosis of infection and initiation of treatment) HIV+TB+ co-infected patients compared to TB infected patients (P value < 0.001) so that the survival rate of HIV+TB+ co-infected patients was shorter than patients infected with TB alone in all of the above mentioned conditions. Thus far in this research, it seems that initiation of symptoms, diagnosis of infection and initiation of treatment are well established predictive factors of survival rate in HIV+TB+ co-infected patients. This may be explained by the much more severe immunodeficiency status of HIV+TB+ co-infected patients.

Under our conditions the survival rate of *tuberculosis*/HIV co-infected patients with positive HAART at one, two, three and five years were 91%, 83%, 66%, 45% and in those with negative HAART, survival rates were 56%, 44%, 32% and 12%, respectively. In contrast, the survival rate of tuberculosis infected patients who underwent treatment at one, two, three and five years were 91%, 82%, 71% and 62%, respectively. A study from Thailand performed on HIV-infected patients with TB showed that the chance of survival after diagnosis of TB infection at one, two, and three years were respectively, 96.1%, 94.0%, and 87.7% for positive HAART patients and 44.4%, 19.2% and 9.3% for those who did not receive HAART (6). Moreover, survival advantage of HAART in the HIV-infected population is probably due to eradication of drug resistance TB in these patients (19). 

A report from Rio de Janeiro indicated that the rates of cure, treatment abandonment, and mortality of TB were 72%, 19% and 6%, respectively (20). Out of 21 patients, 12 (57.1%) patients started HAART while nine (42.9%) patients did not use medication for the treatment of AIDS. Our research showed that the median of survival time in positive HAART patients and in those who did not receive HAART were 48 and 25 months, respectively. Log-rank analysis (log-rank = 5.2) confirmed significant differences between the two groups (P value = 0.03), which prioritize the critical role of monitoring response to treatment.

The limitation of the current study was the low number of co-infected patients. However, the strength of this study was the diagnosis criteria; diagnosis of *M.* *tuberculosis* infection based on a positive TB sputum smear test and the confirmation of HIV positives by the western blotting method. In conclusion, this paper investigates mortality in HIV-associated TB and the predictors of survival and death in patients from a western province of Iran. The current results showed that in HIV-TB+ patients, age, sex and site of TB were significant factors in mortality of patients. This research indicated that the rate of death was 20.7 times greater in HIV/TB co-infected patients than cases infected with TB alone. This appears to be consistent with the literature.

 Overall, the current study shows the necessity for rapid treatment and broader surveillance of co-infection with HIV and tuberculosis especially in infected married individuals, which were more prone to death than single cases. This will include development of further policies, in order to control and reduce the risk of TB/HIV infections. Control of infections by progress in diagnostic and preventive measures such as provision of isoniazid preventive therapy, HIV testing of TB patients, combination of TB and HIV services, control and reduction of the risk of recurrent TB in latent infections will be needed to raise hope for broad surveillance of co-infected patients.
